# Update on Insomnia after Mild Traumatic Brain Injury

**DOI:** 10.3390/brainsci8120223

**Published:** 2018-12-13

**Authors:** Yi Zhou, Brian D. Greenwald

**Affiliations:** 1 Rutgers Robert Wood Johnson Medical School, Piscataway, NJ 08854, USA; yz411@scarletmail.rutgers.edu; 2 JFK Johnson Rehabilitation Institute, Edison, NJ 08820, USA

**Keywords:** mild traumatic brain injury, mTBI, concussion, insomnia, sleep disturbance, treatment

## Abstract

Sleep disturbance after traumatic brain injury (TBI) has received growing interest in recent years, garnering many publications. Insomnia is highly prevalent within the mild traumatic brain injury (mTBI) population and is a subtle, frequently persistent complaint that often goes undiagnosed. For individuals with mTBI, problems with sleep can compromise the recovery process and impede social reintegration. This article updates the evidence on etiology, epidemiology, prognosis, consequences, differential diagnosis, and treatment of insomnia in the context of mild TBI. This article aims to increase awareness about insomnia following mTBI in the hopes that it may improve diagnosis, evaluation, and treatment of sleeping disturbance in this population while revealing areas for future research.

## 1. Methods

**Figure 1:**
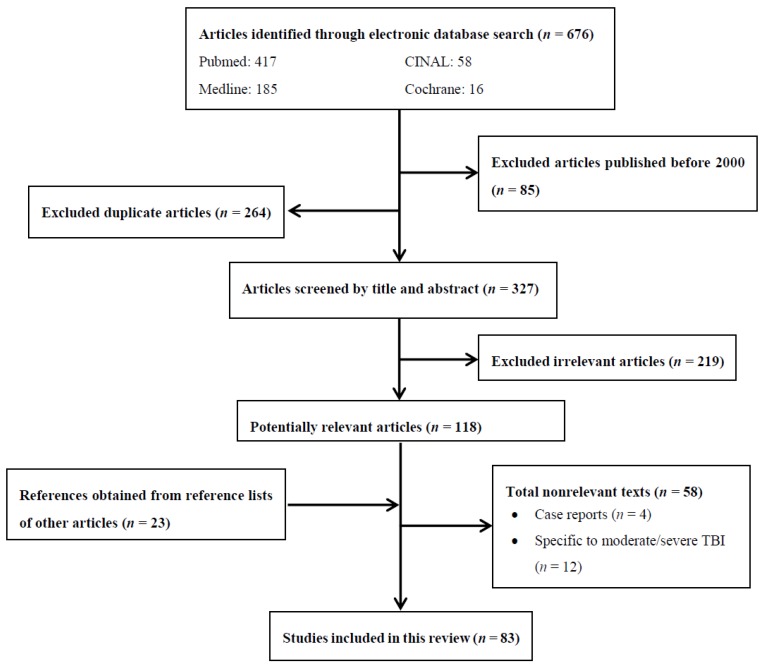
Process flow chart.

References for this narrative review were obtained using a search of online databases PUBMED, MEDLINE, CINAL, and COCHRANE (Figure 1). These online databases were used to search for papers published after the year 2000 using keywords: mild traumatic brain injury, mTBI, concussion, insomnia, sleep disturbance, and treatment. Boolean operator “AND” was used to connect search terms and narrow results. Truncations and MeSH headings were not used. Some references were not identified using the online databases, but were obtained through reference lists of other articles. Our inclusion criteria were studies that looked primarily at brain injury cases, specifically mild traumatic brain injury. Only papers printed in or translated into English were included. We excluded case reports as sources.

## 2. Introduction

Traumatic brain injury (TBI) is a leading cause of disability in the United States. The Center for Disease Control and Prevention recently reported that in 2013 there were 2.8 million cases of TBI-related ER visits, hospitalizations, and deaths [1]. Of the annual cases of traumatic brain injury, the World Health Organization estimates that 70% to 90% of those are mild TBI [2]. The most common groups to sustain a mild TBI are males, teenagers, and young adults, while the most common causes are falls, motor-vehicle collisions, and assault [2]. Despite the high incidence, mTBI numbers are likely an underestimation [1]. One study found that in the emergency department, 56% of cases that qualified as mTBI did not carry a documented diagnosis, suggesting that many patients are potentially undiagnosed [3]. One proposed reason mTBI is frequently missed is that standard imaging in patients with mTBI does not show hemorrhage or other obvious structural abnormalities [4]. Likewise, mTBI-associated sleep disturbances are often unnoticed in the ED and primary care clinics as providers tend to focus on vision changes, nausea, vomiting, head and neck pain [5]. This suggests a need for a higher index of suspicion, targeted questioning and appropriate screening tools to improve diagnosis of both mTBI and associated sleep disturbance [6].

According to the American Congress of Rehabilitation Medicine (ACRM), mild traumatic brain injury is defined as traumatically induced physiologic disruption of brain function that manifests with at least one of the following: any period of loss of consciousness (LOC), memory loss before or after injury, alteration in mental state at time of injury (i.e., feeling dazed, disoriented, confused) or focal neurological deficit(s) that may or may not be transient [7]. Classification as mild requires that severity does not exceed a Glasgow Coma Scale (GCS) score between 13 and 15 after 30 min, a PTA of greater than 24 h, or LOC of more than 30 min [5]. Common symptoms associated with mTBI include headache, dizziness, bad taste, sleep disturbance, nausea/vomiting, impaired balance and coordination, tinnitus, and vision changes [8]. TBI also results in significant cognitive, emotional, and behavioral disorders which all increase morbidity [9]. Poor sleep may develop acutely and last several years post-injury and is described by those who experience a mild TBI as one of the most debilitating consequences [10].

## 3. Pathophysiology of Sleep Disturbance after TBI

Traumatic brain injury is classified into primary and secondary brain injury. Primary injury refers to the structural damage created upon impact. Secondary injury refers to the damage from subsequent cellular processes following primary injury such as excitotoxicity, free radical generation, calcium-mediated damage, hypoxia, and increased intracranial pressure [11,12]. Unsurprisingly, these mechanisms can incur structural, biochemical, and genetic changes implicated in sleep disturbance.

Coup–countrecoup injury typically occurs at the base of the skull in areas of bony prominences so the anterior temporal and inferior frontal regions, including the basal forebrain, are frequently injured. Since the basal forebrain contributes to sleep initiation, injury to this region can lead to insomnia symptoms [8]. The structure of the tentorium has also been associated with sleep pathology. When controlling for cognitive function and mTBI severity, researchers found those with sleep–wake disturbances had longer tentorial lengths and flatter angles, suggesting closer proximity of the tentorial edge to the pineal gland. The authors reason this proximity increases the risk of direct impact between the tentorium and pineal gland subsequently disrupting melatonin pathways [13]. Another study with mTBI patients measured fractional anisotropy (FA) with MRI as a representation of white matter integrity. In patients with sleep disturbance there was reduced parahippocampal FA, which was strikingly similar to findings of early Alzheimer dementia, in which sleep–wake disturbance is one of the earliest symptoms [14]. Using rodent models of TBI, researchers found increased reactive microglia in the thalamus preceding development of sleep disruption. The authors postulate that the inflammatory response may interfere with the thalamocortical network, which regulates sleep–wake patterns [15].

Sleep disturbance after TBI is associated with numerous biochemical changes as well. One study demonstrated lower evening melatonin production in TBI patients compared to healthy controls, which was associated with decreased sleep efficiency, increased wake after sleep onset (WASO), and higher rates of depression and anxiety [16]. Decreased melatonin likely arises from the aforementioned pathway disruptions and/or damage to the suprachiasmatic nucleus [8]. Other reported biochemical associations include changes in levels of hypocretin-1, dopamine, and serotonin, which are neurotransmitters involved in sleep modulation [17]. Decreased levels of IGF-I and testosterone were also found specifically after blast-induced mTBI [18]. More on the distinction of mTBI from blast injury is discussed in Section 5.1.

A variety of genes involved in inflammation, glial function, neuronal plasticity, immunity, and circadian rhythm have implications on sleep disturbance post-TBI [19]. For example, in rodent models several plasticity genes like *Bdnf*, *Homer1a*, and *Fos* have decreased expression a few days after mTBI. This is important because these plasticity genes have demonstrated roles in maintaining sleep homeostasis [19]. Also, in rodent models the astrocyte marker *Gfap* was elevated in the cortex after TBI and prior to the development of sleep disruptions, suggesting that astrocyte activation may contribute to sleep modification [15,19]. In mTBI patients, one of the clock genes, *PERIOD3* (*PER3*), was found to be associated with changes in sleep recovery. Specifically, the *PER3* gene carries a polymorphism that comes in either 4 or 5 tandem repeats and patients who were carriers of 5 repeats interestingly reported improved sleep quality but shorter sleep duration compared to noncarriers at 6 weeks post-mTBI [20].

### Sleep Architecture Changes Seen after mTBI

Sleep architecture refers to the organization and cyclical pattern of normal sleep with specific corresponding electroencephalographic (EEG) activities [21]. Sleep is comprised of slow-wave sleep and paradoxical sleep. Within slow-wave sleep there are 4 stages. Stages 1 and 2 are considered light sleep, while stages 3 and 4 are considered deep sleep. These stages can fall under the term “non-rapid eye movement (NREM) sleep”. The paradoxical sleep stage involves rapid eye movement (REM) sleep which has similar EEG activity to wakefulness and is the stage in which dreaming occurs [21]. In adults, NREM takes up about 20% of the night, while REM takes up about 80% and each stage of sleep is thought to perform independent yet complementary restorative functions [4].

When it comes to sleep architecture after mild TBI, patients generally experience “sleep fragmentation” referring to reduced total sleep time and a greater proportion of sleep in light sleep stages [20]. Observed changes of sleep in mTBI patients include increased stages 1 and 2 sleep and decreased REM sleep [17,22,23]. One study also found no significant difference in sleep and REM latencies in mTBI patients compared to controls [22].

Even though absence of objective findings is common in mTBI patients reporting sleep disturbance, studies recommend that the subjective experience should take precedence [17,22,24]. While mTBI patients with insomnia also tend to underestimate the time spent asleep, subjective sleep is still predictive of depression, anxiety, and general distress [25,26]. As insomnia is by definition a subjective complaint, it is important not to disregard patients even in the absence of objective evidence.

## 4. Insomnia and mTBI

Insomnia is characterized by poor sleep quantity or quality in the forms of delayed sleep onset, nocturnal awakenings with difficulty returning to sleep, waking too early, and not feeling rested despite adequate sleep hours. A required criterion for diagnosing insomnia of any form is that it leads to distress and subjective impairment in the daytime [27]. Insomnia is important to address as it affects patients both psychologically and cognitively throughout recovery and impedes a patient’s return to normal functioning.

Sleep disorders such as insomnia, sleep apnea, narcolepsy, periodic limb movement disorder are all more prevalent in TBI patients compared to the general population [28]. Insomnia is the most commonly reported sleep disturbance with approximately 40%–65% of mTBI patients reporting symptoms of insomnia [12]. However, there are conflicting findings on whether insomnia among TBI patients are over- or underreported. In a survey of 452 patients with TBI, the majority of which were severe TBI (59.9%), 50.2% reported insomnia symptoms, while only 29.4% fulfilled the DSM-IV diagnosis of insomnia [29]. Another study of mTBI patients by Sullivan et al. found that although 42% of their sample had insomnia, only 16% self-reported an insomnia diagnosis [30]. The contrary findings may suggest an association between severity of brain injury and insomnia symptoms, a relationship that will be further discussed in Section 5.2.

A popular model of insomnia contains two components, a general predisposition to developing insomnia followed by an acute stressor [17]. There are various interpretations on how this model applies to TBI. In one interpretation, those suffering from insomnia post-TBI may have had previous episodes of insomnia or a family history. The acute stressor would therefore be the TBI itself [17]. Another interpretation surmises that both aspects of the model may be affected by TBI because pathophysiological changes of the brain create a predisposition for insomnia while acute stressors come in the forms of comorbidities such as pain, depression, and anxiety [11,18]. TBI patients will also often experience multiple psychosocial stressors simultaneously such as inability to return to work, financial difficulties, redefined familial roles, and strained social relationships. Increased familial discord, marital problems, and litigation procedures have been documented with TBI patients and are obvious sources of significant stress [31]. The patient experience and environment are therefore important to consider in management of these patients.

## 5. Epidemiology of Insomnia after mTBI

Within the general population, sufferers of insomnia are more likely to be white, older, female, and unmarried. Although prevalence of insomnia symptoms increases with age in the general population, sleep dissatisfaction and diagnoses were found to be independent of age [32]. The demographic characteristics differ in the context of TBI as a study by Fichtenberg et al. of patients with various severities of TBI found no relationship between insomnia and gender, age, or education [33]. In the mTBI population, age at time of injury may have implications on the development of insomnia. For example, adolescence is a time of increased risk for sleep problems due to physiologic changes and increased societal and academic demands. Thus, when adolescents experience a mild TBI they have a higher predispositon than younger children to develop chronic sleep problems [34]. It is generally believed that TBI outcomes like sleep disturbance worsen with increased age possibly due to reasons such as decreased ability to compensate, decreased cerebral reserve, and pre-existing comorbidities [35]. When it comes to mTBI, literature remains divided on how age affects outcomes [35]. Some studies suggest increased symptom severity with age; however, a study by Hu et al. found that middle-aged mTBI patients (36–55 years) had a higher severity of sleep disturbance compared to elderly patients [35]. The authors suggest that this finding may be due to middle-aged patients noticing more significant deviations from their baseline functional status and experiencing more daily stressors such as employment and living with dependents. Although studies are revealing potential risk factors, further research is needed to better comprehend the risk factors that predispose and precipitate insomnia after mild TBI.

### 5.1. Repeat TBI and Blast Injury

Repeat mTBIs are found to increase the likelihood and severity of insomnia as well as cause persistent deficits in spatial learning and memory, subacute anxiety, and depression relative to the aftermath of a single mTBI [36]. A study of military personnel found that 20% of patients with a single mTBI reported insomnia, whereas 50% of patients who experienced multiple mTBIs reported insomnia [37]. It is worth noting that blast injury accounts for around 60% of military-related TBIs of which 80% are classified as mild TBI [38]. Unlike blunt trauma associated with other forms of TBI, blast injury produces shockwaves that create propagating pressure transients that can lead to diffuse axonal injury, contusion, edema, and hemorrhage [39]. This describes only primary blast injury and it is also important to consider potential damage from shrapnel, thermal effects of detonation, and psychological consequences [39]. In a broad context, TBI and blast-related TBI (bTBI) are often discussed in the same vein because evidence points to similar impairments after injury. One study comparing bTBI to other causes of brain injury found no significant differences in sleep impairment along with cognitive impairment, pain, and other symptoms post-injury [40]. However, distinct consequences of bTBI include hearing loss, tinnitus, and increased incidence of post-traumatic stress disorder (PTSD) symptoms [39]. Therefore, consequences of blast-related TBI compared to those from other forms of TBI are not synonymous and differences are important to keep in mind in practice and literature. Regarding repeat TBIs, sleep disturbance is itself a risk factor for repeat brain injury. Insomnia is known to increase the risk of industrial and vehicle accidents, thereby increasing risk of TBI and reoccurrence [27]. One survey found that chronic insomnia doubled the risk of automobile accidents due to sleepiness [41]. Since insomnia after TBI increases risk for reoccurrence, safety precautions and follow-up should be emphasized.

### 5.2. Implications of TBI Severity

A frequently mentioned and somewhat counterintuitive finding is that mild TBI is more strongly associated with insomnia and other sleep disturbances compared to more severe TBI [35]. Opposition to this correlation includes a prospective study by Baumann et al., which found that severity of inciting head injury did not predict sleep–wake disturbances [42]. Despite this finding, evidence generally supports the relationship between insomnia and milder TBI [29,33,43]. The higher prevalence of insomnia in mTBI patients may be secondary to increased awareness of impairment and disability leading to an increase in self-reporting [33]. Mild TBI patients may also be under more pressure to reintegrate into normal life which could lead to increased stress and sleep issues [8,35].

## 6. Prognosis

Prognosis of mTBI is typically good with one study finding that almost all previously healthy adults (96%) return to work/normal activities within one year post-mTBI [44]. Despite this prognosis, a large prospective cohort study discovered a that significant minority of patients after mild TBI, around 20%, are not functionally recovered even by one year post-injury [45]. Sleep difficulty is one of the postconcussive symptoms that is assumed to resolve spontaneously over time. Along with depression and anxiety, all are expected to improve; however, one study found that only sleep quality improved to the pre-mTBI level by 6 weeks [46]. This implies that recovery from sleep disturbance is relatively rapid, yet studies that extend beyond the subacute stage suggest otherwise. For example, a study found that for veterans with mTBI, poor sleep quality lasted on average 6 years post-injury and was independent of combat exposure, PTSD, mood disorders, anxiety, and substance use [47].

It is generally accepted that negative outcomes of mTBI resolve even more rapidly within the younger population with complete recovery expected within 2–3 months for children and adolescents [34]. However, a longitudinal study of children between the ages of 8–16 years found little improvement in sleep difficulties after 6 months and 28% still experienced poor sleep at one year post-mTBI [48]. In the study, sleep quality at 1 month was predictive of symptom severity and behavioral outcomes at 12 months, suggesting that prompt intervention may facilitate recovery [48]. Persistent sleep impairment among the younger population is reinforced with another study that found that around a third of adolescents after mTBI had greater levels of sleep disturbance compared to healthy peers even 6 years after injury [49]. These findings appear to counter the common belief of mTBI’s speedy recovery especially in younger patients. While other symptoms post-mTBI may resolve, these study findings (further detailed in Table 1) suggest that sleep disturbance persists for years in a significant proportion of patients regardless of age group.

### Consequences of Sleep Impairment

Sleep quality impacts all areas of daily functioning and is also crucial for the recovery process. Memory, attention, and executive functions are the cognitive domains most affected by sleep impairment [51]. This aligns with recent evidence demonstrating the importance of sleep on neural growth and plasticity, learning, and memory consolidation [4]. Poor sleep was found to be significantly predictive of poorer post-concussion symptoms, mood, community integration, and cognitive ability at one year post-injury [52]. Sleep disruption may also act as a cellular stressor and lead to cognitive decline. Studies found that disrupted sleep leads to accumulation of hyperphosphorylated tau and amyloid beta plaque accumulation from oxidative stress [23].

Fatigue is a common sequelae post-mTBI that emerges as soon as a few days post-injury [53]. There exist two supported models of cognitive fatigue after mTBI, including fatigue secondary to increased work to process information and fatigue secondary to impaired sleep [54]. One study found the prevalence of fatigue to be 68% at one week post-mTBI and decrease to 38% after 3 months [55]. Although fatigue is found to be higher at the 4 month mark in mTBI compared to sTBI, fatigue tends to reduce over time after mild TBI and increase after severe [53]. However, even by 6 months after sustaining an mTBI, 32%–34% of individuals reported fatigue [55,56]. The concern regarding the high prevalence of fatigue in the mTBI population is association with higher reported levels of functional impairment, depression, and cognitive difficulties [53,56].

For adolescents, studies find that persistent sleep impairment following mTBI is associated with poorer quality of life, greater depressive symptoms and decreased participation in normal roles [34]. In a study of university students (18–25 years) with mTBI, sleep impairment led to increased daytime dysfunction along with lower levels of enthusiasm and energy in completing tasks. Participants also experienced behavioral problems which were moderately correlated with sleep-related daytime dysfunction in the forms of social withdrawal, poor relationships, clumsiness, and speech difficulties [57]. The consequences of poor sleep in these populations are particularly detrimental during a time when social integration, academic functioning, and development are critical.

Within the working population, individuals with sleep impairment present with greater absenteeism, increased presenteeism, lower job satisfaction and work productivity loss [27,41]. In a study of workers with delayed recovery from mTBI, insomnia was the only variable associated with greater odds of disability while age, sex, education, income, and marital status were not associated with greater perceived disability [58]. Therefore, prioritizing sleep management in these patients may expedite return to normal functioning and mitigate potential sources of stress.

## 7. Differential Diagnosis of Sleep Disturbance after TBI

### 7.1. Pain

Pain is one of the most frequent complaints post-mTBI and along with insufficient management is a significant contributor to sleep disturbance [59]. Comorbid pain was found in over 60% of mTBI patients and often presented in the forms of headaches, joint, neck, shoulder and back pain [59,60,61]. Similar to the trends of sleep disturbance, reports of pain are more frequent in the mTBI population than in more severe injuries. A possible explanation is that patients with severe injuries are often bedridden and treated with paralytic agents allowing healing of cervical injuries. Those with mild injuries may continue to use those damaged muscles and ligaments, thereby interfering with healing [62]. Those with severe injuries may also have more difficulty communicating their pain to providers [62]. In early recovery, Suzuki et al. observed that pain increased sleep need to over 8 h in a third of mTBI patients [60]. In mTBI patients with pain, fast beta and gamma electroencephalographic activity were observed in frontal, central, and occipital electroencephalographic (EEG) within all sleep stages. This finding suggests that the increased need for sleep is secondary to persistent wake EEG activity, leading to unrestful sleep [59]. Evaluation of pain is a necessary precursor to the management of new sleep complaints after mTBI as adequate pain management may treat insomnia as well.

### 7.2. Sleep Apnea

Behind insomnia, sleep apnea is one of the most frequently diagnosed sleep disorders post-TBI [28]. Both obstructive and central sleep apneas are more prevalent in the TBI population and seem to arise from a complex interaction between brain injury, decreased arousal, and impaired respiratory effort [6,63]. Obstructive sleep apnea (OSA) refers to intermittent episodes of upper airway obstruction that reduces blood oxygenation [28]. Studies have demonstrated prevalence of obstructive sleep apnea to be 25% to 35% following TBI of any severity which is substantially higher than general population findings [63].

While many patients complain of insomnia and will lack objective findings, patients with diagnosable sleep apnea will often fail to recognize the problem and only describe poor day time vigilance [28]. Although costly and time-consuming, the gold standard for diagnosing sleep apnea is polysomnography (PSG), which will commonly find increased sleep onset latency, poor sleep efficiency, and decreased REM latency [6]. Sleep apnea is predictive of higher all-cause mortality and recurrent vascular events after stroke and TIA implying urgency in diagnosing and treating sleep-disordered breathing within the TBI population [6]. It is worth noting that studies that found sleep apnea of any severity significantly increases risk of TBI [64]. Therefore, sleep apnea may also be an important target in the prevention of TBI. Treatment is unchanged to those with other causes of sleep-disordered breathing where positive airway pressure (PAP) is the standard of care [4].

### 7.3. Post-Traumatic Stress Disorder

The majority of studies on the relationship between post-traumatic stress disorder (PTSD) and traumatic brain injury understandably involve veterans where the rate of PTSD after TBI ranges from around 27% to 44% [65]. Among the civilian population suffering from nonmilitary trauma, one study found that 14% to 56% of TBI patients had comorbid PTSD [65]. PTSD is diagnosed via criteria released by the American Psychiatric Association which shares many symptoms with TBI, often causing attribution of symptoms to be difficult [66]. One of the shared and core symptoms of PTSD is sleep disturbance, found in around 70% of PTSD patients [12]. Although it is difficult in PTSD–TBI comorbid patients to assess the roles each play in sleep impairment, certain sleep pathology may be distinguishable between the two. One study comparing TBI and PTSD patients among returning veterans found no differences in the rates of OSA, excessive awakenings and daytime sleepiness but on PSG, PTSD patients demonstrated greater arousal frequency while TBI patients demonstrated greater slow-wave sleep [67].

Similar to symptoms, the treatment of sleep disturbance in PTSD and TBI patients overlaps. Despite similarities, certain treatments may be prioritized depending on the presence of comorbid PTSD with TBI. VA/DOD Clinical Practice Guidelines for the treatment of PTSD recommend prazosin, an alpha-1 adrenergic antagonist, as treatment for sleep impairment and nightmares in PTSD [65,66]. Other supported pharmacologic options for PTSD include venlafaxine, a serotonin norepinephrine reuptake inhibitor (SNRI), and selective serotonin inhibitors such as sertraline and paroxetine [65]. Likewise, cognitive behavioral therapy (CBT) is a mainstay of treatment for sleep impairment in TBI but studies recommend trauma-focused CBT for comorbid PTSD in reducing symptoms [65]. For these patients, there is likely a complex interplay between TBI and PTSD that results in sleep impairment and currently cannot be separated into exclusive contributions. Therefore, more research is needed in studying the associations between sleep disturbance and TBI patients with comorbid PTSD. Recommended management of sleep disturbance in the PTSD population differs slightly from TBI and is therefore up to clinical judgment for attempting PTSD targeted treatments.

### 7.4. Circadian Rhythm

Circadian rhythm disorders are sometimes mistaken for insomnia post-TBI. Circadian rhythm disorder refers to disruption of the normal 24 hr cycle of body patterns such as body temperature and melatonin secretion [10,23]. Circadian sleep disturbances come in the forms of irregular sleep–wake pattern and more commonly delayed sleep phase syndrome [68]. A study by Ayalon et al., using actigraphy, salivary melatonin, temperature measurement, and polysomnography, found that 36% of patients who were diagnosed with insomnia actually had circadian sleep disturbance [68]. The distinction between insomnia and circadian rhythm disorder is important as treatment differs. Rather than prescribe hypnotics, melatonin or bright light therapy are more appropriate for these patients [68].

## 8. Treatment

Many treatment options are available for patients suffering from insomnia post-TBI (Table 2). Despite this, insomnia is often undertreated and patients seek treatment independent of health care providers in the forms of over-the-counter (OTC) medications and alcohol [69]. In the context of TBI, one study found that around 60% of those fulfilling diagnosis of insomnia were left untreated [29].

Similar to the management of most chronic medical conditions, providers should begin with conservative measures and proceed to more aggressive options only when necessary. As was discussed, this begins with ruling out common comorbidities as causes of sleep impairment. After other etiologies are considered, further interventions can be sought.

### 8.1. Sleep Hygiene

Sleep hygiene is a broad term that refers to adjustments that improve sleep health. The basis is to replace stimulating behaviors with sleep-promoting behaviors. Examples of adjustments include exercising, consuming a snack before bed, keeping the bedroom dark, limiting noise, maintaining a regular sleep schedule, and reducing intake of stimulants and alcohol [70]. Individuals with chronic insomnia will often spend more time in bed and nap during the day leading to irregular sleep–wake schedules. These behaviors likely desynchronize the natural cycle and contribute to sleep disturbances [31]. Many studies advocate sleep hygiene incorporation into the care plans of TBI patients as it is low cost, low risk, and noninvasive [71]. Studies have demonstrated that increased knowledge of sleep hygiene post-TBI was associated with better sleep habits and subsequent sleep quality improvements [72].

### 8.2. Cognitive Behavioral Therapy & Digital CBT

Cognitive behavioral therapy (CBT) encompasses sleep hygiene along with other techniques such as relaxation, sleep restriction, and stimulus control. The goal of CBT is to reduce unrealistic expectations and anxiety towards sleep by identifying and mitigating deleterious thoughts surrounding and during sleep [70]. A key component of CBT is the sleep diary that includes self–reported data on time in bed, medication use, caffeine intake, exercise, and awakenings as an attempt to eliminate recall bias [70]. A systematic review by Bogdanov et al. demonstrated significant improvements in insomnia severity and sleep diary data after CBT among patients with TBI and comorbid insomnia. The benefits of CBT were found to appear within 1–2 weeks of implementation and sustained by time of follow-up 3 months later [73]. Another study found that patients undergoing CBT also had significant improvements in both fatigue and depression that persisted at follow-up 2 months later [74]. These findings support why CBT is widely advocated as a standard of care for insomnia including within the TBI population.

“Digital CBT” has received growing interest in recent years and is a general term referring to CBT provided via web and mobile platforms [75]. A recent RCT comparing dCBT to sleep hygiene counseling for general insomnia found small improvements in functional health and psychological well-being, but large improvements in sleep-related quality of life and insomnia symptoms [76]. When comparing dCBT to CBT, meta-analyses demonstrate that the effects of dCBT on insomnia at increasing total sleep time, decreasing sleep onset latency and decreasing WASO are in the ranges of conventional CBT, suggesting similar effectiveness [75] Relative to CBT, dCBT also tended to be more cost-effective with lower societal and healthcare costs [75]. Besides similar efficacy and improved cost-effectiveness, dCBT’s ability to disseminate effective treatment for insomnia is likely its greatest advantage [75]. Although dCBT appears to be a promising avenue for treatment, much research is needed on the effects of dCBT for insomnia post-TBI.

## 9. Pharmacologic Options

### 9.1. Benzodiazepines/Z-Drugs

Benzodiazepines such as diazepam, lorazepam, and alprazolam have largely fallen out of use due to side effects such as dependency, daytime sedation, cognitive impairment, and increased risk of falls/accidents that are deleterious to recovery from TBI [77]. The Z-drugs (zapleplon, zopliclone, and zolpidem) are alternatives. Similar to benzodiazepines, Z-drugs are also GABA agonists but act more specifically on the type 1 receptor [78]. Z-drugs are not without their potential side effects, including daytime sedation and sensory distortions, but generally have no daytime consequences on cognitive and psychomotor function. Z-drugs also have shorter half-lives compared to benzodiazepines ranging from 1 hr with zapleplon to 5 hrs with zopliclone. It is believed that the selective target and faster offset of action contribute to the preferable side effect profile of Z-drugs as compared to benzodiazepines [78]. While Z-drugs have a significantly lower incidence of dependence relative to benzodiazepines, there is still a risk of abuse especially in patients with a history of substance abuse, dependence, or psychiatric diseases [79]. Research on the efficacy of Z-drugs within the TBI population is limited. One study comparing the efficacies of lorazepam and zopiclone in treating insomnia for stroke and brain injury patients found that both groups slept more than 7 h and did not differ in quality of sleep, suggesting similar effectiveness [80]. Long-term therapeutic benefits of both benzodiazepines and Z-drugs are limited, so while indicated for short-term use, one should defer to attempting non-pharmacologic treatments.

### 9.2. Trazodone

Trazodone is a heterocyclic antidepressant and one of the most frequently prescribed drugs for treatment of insomnia in TBI patients [78]. Its mechanism in humans remains poorly understood; however, animal models have shown that trazodone inhibits serotonin re-uptake [78]. For TBI patients, trazodone is generally well tolerated and unlike the tricyclic antidepressants, has little to no anticholinergic effect [81]. Compared to Z-drugs, trazodone has a relatively longer half-life at 5–9 h, so daytime sedation is a potential side effect [81]. Despite trazodone’s usage, there are no clinical trials on trazodone for insomnia treatment in the TBI population. For insomnia in the general population, trazodone increased sleep duration compared to placebo and in patients with comorbid depression was also found to increase total sleep time [78]. As depression is a common comorbidity in patients with TBI, trazodone may be first line for select patients.

### 9.3. Melatonin/Melatonin Agonists

Melatonin is a hormone synthesized in the pineal gland that is triggered by the absence of light and plays a crucial role in the sleep–wake cycle. As mentioned, TBI patients tend to have disruptions in the melatonin pathways, which result in lower melatonin levels later in the day compared to healthy controls [16]. A RCT comparing the efficacy of melatonin supplementation to placebo for TBI patients with sleep disturbance found improved sleep quality, sleep efficiency, and decreased anxiety with no significant difference in sleep latency [82].

Efficacy of exogenous melatonin has led to the development of melatonin agonist ramelteon. Similar to melatonin supplementation, ramelteon has a superior side effect profile [78]. In a pilot study investigating the effects of ramelteon on sleep among patients with TBI, patients were given 8 mg nightly over 3 weeks. The study demonstrated a significant increase in total sleep time and a modest improvement in sleep latency compared to placebo. On standardized neuropsychological testing, participants also had improved scores particularly in executive functioning [83]. Currently, no generic formulation is commercially available so as melatonin agonists increase in popularity, they may become more affordable and viable options.

## 10. Summary and Conclusions

Insomnia is a highly prevalent and debilitating condition within the mTBI population with around 40%–65% of patients reporting symptoms of insomnia. As both mTBI and associated insomnia are underdiagnosed, a proper history and high clinical suspicion are important in detecting underlying sleep disorder. Although most patients with mTBI return to normal functioning, sleep disturbance is a subtle and often persistent condition lasting many years for a significant proportion of patients. Since many brain injury patients self-treat and feel lost when addressing these new sleep disturbances, it is important for providers to follow up with patients regarding sleep post-TBI and maintain a low threshold to intervene as poor sleep hinders recovery and social reintegration. When addressing impaired sleep, rule out common comorbidities prior to management as treatment may differ. Pharmacologic options are effective for situational use but may have negative consequences in the long term. As sleep impairment may last years after injury, attempting nonpharmacologic treatments such as sleep hygiene counseling and CBT are preferred as they demonstrate persistent benefits. Since mild TBI is by far the most common severity of TBI and data suggests that milder brain injury increases risk of insomnia, more research is needed towards studying insomnia and its treatment within the mild traumatic brain injury population.

## Figures and Tables

**Table 1:** Summary of study findings of sleep disturbance chronicity after mild traumatic brain injury (mTBI).

Study	Study Design	Participants	Sleep Outcome Measure	Results	Limitations
Ma et al. (2014) [46]	Prospective cohort	mTBI group: ⋅ *n* = 100 ⋅ Age: >20 years, (mean = 38.88 years) Control group: ⋅ *n* = 137, ⋅ Age: >20 years (mean: 29.86 years) ⋅ without TBI	Pittsburgh Sleep Quality Index (PSQI)	⋅ Baseline mTBI PSQI scores significantly different from scores of control ⋅ At 6 weeks follow-up, mTBI PSQI scores improved significantly and were not significantly different from scores of control	⋅ Medication use may have interfered with assessment ⋅ Unidentified pre-existing comorbidities ⋅ Only evaluated subacute stage of sleep quality ⋅ Self-report
McMahon et al. (2014) [45]	Prospective cohort	mTBI study population: ⋅ *n* = 375 ⋅ Age: >18 years, (mean = 44 years)	Postconcussion Syndrome (PCS) Symptom Checklist	⋅ At 3 months, 50.2% of mTBI patients report at least one sleep symptom (*n* = 348) ⋅ At 1 year follow-up, 53.5% of mTBI patients report at least one sleep symptom (*n* = 199)	⋅ No control group ⋅ Loss of patients to follow-up ⋅ Did not analyze contribution of medical history on outcome ⋅ Self-report
Martindale et al. (2017) [47]	Cross-sectional	mTBI study population: ⋅ Veterans (*n* = 527) ⋅ Age: >21 years, (mean = 35.47 years)	Pittsburgh Sleep Quality Index (PSQI)	⋅ 56.2% of sample reported clinically significant poor sleep quality ⋅ Poor sleep quality lasts on average 6 years independent of combat exposure, post-traumatic stress disorder (PTSD), mood disorders, anxiety disorders, and substance use disorders	⋅ Deployment related-mTBI limits generalizability ⋅ Cross-sectional data, unable to evaluate temporal relationship ⋅ Self-report
Theadom and Parag et al. (2016) [50]	Longitudinal population study	mTBI study population: ⋅ *n* = 341 ⋅ Age: >16 years, (mean = 37.5 years)	Rivermead Post Concussion Symptoms Questionnaire (RPQ)	⋅ 43% of sample reported sleep disturbance at baseline ⋅ At 12 months 32% still reported sleep disturbance	⋅ Lack of information on prior mood, psychiatric and medical conditions ⋅ Self-report
Theadom and Starkey et al. (2016) [48]	Prospective cohort	mTBI group: ⋅ *n* = 109 ⋅ Age: 8–16 years, (mean = 11.49 years) Control group: ⋅ *n* = 68, ⋅ Age: 8–16 years (mean: 11.52 years) ⋅ Without TBI	Pittsburgh Sleep Quality Index (PSQI)	⋅ At 12 months, 28% of mTBI group reported poor sleep quality compared to 39% at 1 month ⋅ mTBI group had significantly poorer sleep quality compared to healthy controls (OR = 3.09) at 12 months post-injury	⋅ Data from parent reporting
Pillar et al. (2003) [49]	Cross-sectional	mTBI group: ⋅ *n* = 98 ⋅ Age: 8–18 years, (mean = 13.5 years) ⋅ 0.5–6 years post-injury at time of study Control group: ⋅ *n* = 80, ⋅ Age: 8–18 years (mean: 12.4 years) ⋅ Without TBI	Study designed questionnaire	⋅ 28% of mTBI group reported long-term sleep disturbance (6 months to 6 years) compared to 11% of controls	⋅ Did not stratify findings based on mTBI chronicity ⋅ Cross-sectional data, unable to evaluate temporal relationship ⋅ Self-report ⋅ Response rate (98/150) may inflate prevalence

**Table 2:** Pros/Cons of Commonly Used Treatments for Insomnia after TBI.

Intervention	Pros	Cons
**Sleep Hygiene Counseling**	⋅ Effective in improving sleep quality and reducing daytime sleepiness ⋅ Inexpensive, low risk, noninvasive ⋅ Persistent improvement on sleep ⋅ Recommendations may be catered to patient environment	⋅ Variable recommendations, provider-dependent ⋅ Issue of non-compliance ⋅ Patients may be non-receptive to indirect intervention ⋅ Barriers may exist for implementing changes (ex. living arrangement, occupation, economic status, disability, dependents, etc.)
**Cognitive Behavioral Therapy (CBT)**	⋅ Effective in improving insomnia severity, sleep efficiency, and quality ⋅ Includes sleep hygiene counseling ⋅ Low risk, noninvasive ⋅ Benefits appear within 1–2 weeks ⋅ Persistent improvement on sleep ⋅ Persistent improvement in comorbid fatigue, depression and anxiety ⋅ Newer digital CBT may address accessibility and scalability	⋅ Time commitment (meetings, maintaining sleep diary) ⋅ Issue of noncompliance ⋅ Financial costs ⋅ Provider dependent efficacy ⋅ Variable settings (one to one or in group settings)
**Benzodiazepines** (i.e., flurazepam, lorazepam, estazolam)	⋅ Effective in increasing total sleep time and improving sleep quality	⋅ Risk of dependency and abuse ⋅ Associated with daytime sedation and cognitive impairment ⋅ Increases risk of falls/accidents ⋅ Short-term benefit
**Z-drugs** (i.e., zaleplon, zolpidem, zopiclone)	⋅ Effective in increasing total sleep time and improving sleep quality ⋅ Well tolerated, no daytime cognitive or psychomotor impairment ⋅ Significantly lower incidence of dependence compared to benzodiazepines	⋅ Associated with daytime sedation ⋅ Potential psychological dependency and abuse potential ⋅ May cause sensory distortions ⋅ Short term benefit ⋅ Lack of research in TBI patients
**Trazodone**	⋅ Increases sleep duration in insomnia and with comorbid depression ⋅ Generally well tolerated ⋅ Comparable antidepressant effect to selective serotonin reuptake inhibitors (SSRI) and tricyclic antidepressants(TCA) ⋅ Decreased anticholinergic effects compared to TCA	⋅ Associated with daytime sedation, headache, dry mouth, sexual dysfunction, orthostatic hypotension ⋅ Short-term benefit ⋅ Lack of research in TBI patients
**Melatonin & Melatonin Agonists** (i.e., ramelteon, tasimelteon)	⋅ Increases total sleep time and decreases sleep latency ⋅ OTC melatonin affordable and accessible ⋅ No risk of dependency or tolerance	⋅ Agonists are costly ⋅ Short-term benefit

Available pharmacologic and non-pharmacologic interventions for treatment of insomnia. [70,71,72,73,74,75,76,77,78,79,80,81,82,83].
